# Supplementary material for: Lipids and Transaminase in Antiretroviral-Treatment-Experienced People Living with HIV, Switching to a Doravirine-Based vs. a Rilpivirine-Based Regimen: Data from a Real-Life Setting
Source: Viruses. 2023 Jul 23;15(7):1612. doi: 10.3390/v15071612 (PMC10383194; doi:10.3390/v15071612)
Supplement: Supplementary file 1 [file viruses-15-01612-s001.zip › viruses-2478901-supplementary.pdf]

Table S1. Other DOR-based regimens (n=114).

regimen	N	%
DOR/DTG/	50	43.86
FTC/TAF/DOR/	22	19.30
DOR/RAL/	10	8.77
DOR/DRV/COBI/	8	7.02
3TC/DOR/DTG/	7	6.14
3TC/ABC/DOR/	4	3.51
FTC/TAF/DOR/BIC/	3	2.63
FTC/DOR/TDF/	2	1.75
3TC/ABC/NVP/DOR/	1	0.88
3TC/DOR/RAL/	1	0.88
DOR/DRV/RTV/	1	0.88
DOR/RAL/MRV/	1	0.88
DOR/TDF/DRV/COBI/	1	0.88
DOR/TDF/DTG/	1	0.88
FTC/TAF/DOR/DRV/COBI/	1	0.88
FTC/TAF/DOR/DTG/	1	0.88

Table S2. Baseline characteristics of PLWH on 3TC/TDF/DOR and other DOR-based regimens.

	DOR-based regimens				
	3TC/TDF/DOR		Other DOR-based		P
	N=142	55.5%	N=114	44.5%	
Sex					
F	36	25.4	37	32.5	
M	106	74.6	77	67.5	0.21
Ethnicity					
Caucasian	120	84.5	106	93.0	
Other	22	15.5	8	7.0	0.04
Risk factor					
IVDU	14	9.9	23	20.2	
Other	22	15.5	19	16.7	
Sexual	106	74.6	72	63.2	0.05
HBsAg					
	16	11.3	15	13.2	
N	115	81.0	98	86.0	
Y	11	7.7	1	0.9	0.01
HCV					
	14	9.9	11	9.6	
N	115	81.0	72	63.2	
Y	13	9.2	31	27.2	0.0001
Previous PI					
N	123	86.6	69	60.5	
Y	19	13.4	45	39.5	<0.0001
Previous NNRTI					
N	74	52.1	75	65.8	
Y	68	47.9	39	34.2	0.03
Previous INI					
N	89	62.7	41	36.0	
Y	53	37.3	73	64.0	<0.0001
Lipid-lowering drugs					
N	115	81.0	84	73.7	
Y	27	19.0	30	26.3	0.16

	DOR-based regimens				
	3TC/TDF/DOR		Other DOR-based		P
	N=142	55.5%	N=114	44.5%	
Diabetes					
N	138	97.2	105	92.1	
Y	4	2.8	9	7.9	0.09
AST >40 IU/L					
N	126	88.7	102	89.5	
Y	16	11.3	12	10.5	0.85
ALT >40 IU/L					
N	126	88.7	102	89.5	
Y	16	11.3	12	10.5	0.85

Table S3. Baseline characteristics of PLWH on 3TC/TDF/DOR and other DOR-based regimens.

	DOR- based regimens				
Baseline	3TC/TDF/DOR		Other DOR-based		P
	Mean	SD	Mean	SD	
Age, years	49.0	10.9	55.8	10.8	<0.0001
BMI, Kg/m^3	26.5	5.7	25.9	4.9	0.38
Weight, Kg	78.6	16.3	74.4	14.3	0.04
HDL-C, mg/mL	48.5	13.2	50.6	15.7	0.25
Total Cholesterol, mg/mL	199.0	42.8	194.0	48.0	0.38
LDL-C, mg/mL	121.4	37.2	112.7	39.3	0.08

Table S4. Baseline characteristics of PLWH on 3TC/TDF/DOR and other DOR-based regimens.

	DOR-based regimens						
	3TC/TDF/DOR			Other DOR-based			P
	Median	Q1	Q3	Median	Q1	Q3	
CD4, cells/mm^3	756.0	510.0	970.0	670.0	496.0	1012.0	0.43
Triglycerides, mg/mL	121.0	84.0	173.0	122.0	98.0	180.0	0.38
TC/HDL-C, mg/mL	4.2	3.4	5.1	3.8	3.3	4.6	0.04
AST, IU/L	22.0	18.0	28.0	22.0	18.0	27.0	0.48
ALT, IU/L	23.0	18.0	30.0	21.5	16.0	31.5	0.78

Table S5. Baseline characteristics of PLWH on 3TC/TDF/DOR and other DOR-based regimens, not on lipid-lowering drugs (n=199).

	DOR-based regimens				
	3TC/TDF/DOR		Other DOR-based		P
	Mean	SD	Mean	SD	
HDL-C, mg/mL	48.7	12.5	50.6	15.3	0.36
Total cholesterol, mg/mL	196.5	42.1	196.9	49.9	0.95
LDL-C, mg/mL	120.7	37.8	116.3	39.1	0.44
	Median	IQR	Median	IQR	
TC/HDL-C	4.2	3.4-4.8	4.0	3.3-4.7	0.34
Triglycerides, mg/mL	113	80-168	116	90-174	0.27

Table S6. Baseline characteristics of 401 experienced men enrolled in the SCOLTA Project.

	Cohort				
	Doravirine		Rilpivirine		P-value
	N or mean or median	% or SD or IQR	N or mean or median	% or SD or IQR	
Age, years, mean, SD	52.4	11.7	46.8	9.7	<0.0001
BMI, Kg/m^2, mean, SD	26.0	4.5	24.8	3.5	0.005
Weight, Kg, mean, SD	79.1	14.1	76.0	13.1	0.02
Ethnicity, n, %					
Caucasian	168	91.8	205	94.0	
Other	15	8.2	13	6.0	0.38
Risk factor for HIV acquisition, n, %					
IDU	28	15.3	42	19.3	
Other	28	15.3	12	5.5	
Sexual	127	69.4	164	75.2	0.04
HbsAg, n, %					
Missing	19	10.4	7	3.2	
Negative	155	84.7	196	89.9	
Positive	9	4.9	15	6.9	0.52
HCV, n, %					
Missing	15	8.2	1	0.5	
Negative	136	74.3	166	76.1	
Positive	32	17.5	51	23.4	0.29
HbsAg and/or HCV, n, %					
Missing	14	7.7	1	0.5	
Negative	129	70.5	154	70.6	
Positive	40	21.9	63	28.9	0.24
HCVRNA, n, %					
Undetectable	36	90.0	28	44.4	
Detectable	4	10.0	35	55.6	<0.0001
HIVRNA, n, %					
Undetectable	166	90.7	207	95.0	
Detectable	17	9.3	11	5.0	0.10

	Cohort				
	Doravirine		Rilpivirine		P-value
	N or mean or median	% or SD or IQR	N or mean or median	% or SD or IQR	
Previous PI, n, %					
N	140	76.5	120	55.0	
Y	43	23.5	98	45.0	<0.0001
Previous NNRTI, n, %					
N	107	58.5	114	52.3	
Y	76	41.5	104	47.7	0.21
Previous INSTI, n, %					
N	92	50.3	204	93.6	
Y	91	49.7	14	6.4	<0.0001
Current TDF, n, %					
N	76	41.5	14	6.4	
Y	107	58.5	204	93.6	<0.0001
Previous TDF, n, %					
N	149	81.4	39	17.9	
Y	34	18.6	179	82.1	<0.0001
CD4, cells/mm^3, median, IQR	696	494-941	640	495-811	0.02
CDC stage, n, %					
missing	3	1.6	.	.	
A	81	44.3	117	53.7	
B	59	32.2	55	25.2	
C	40	21.9	46	21.1	0.17
TC, mg/dL, mean, SD	191.9	45.6	188.9	43.0	0.50
HDL-C, mg/dL, mean, SD	45.8	10.6	43.8	11.0	0.07
LDL-C, mg/dL, mean, SD	115.6	39.9	113.4	39.0	0.58
TC/HDL-C	4.2	3.6-5.1	4.4	3.6-5.2	0.23
Triglycerides, mg/dL, median, IQR	129	91-181	129	89-186	0.90
Lipid lowering drugs, n, %					
N	138	75.4	197	90.4	
Y	45	24.6	21	9.6	<0.0001

	Cohort				
	Doravirine		Rilpivirine		P-value
	N or mean or median	% or SD or IQR	N or mean or median	% or SD or IQR	
Diabetes, n, %					
N	173	94.5	207	95.0	
Y	10	5.5	11	5.0	0.85
Blood glucose, mg/dL, mean, SD (non-diabetics)	91	17	91	12	
AST, IU/dL, n, %					
≤40	173	94.5	180	82.6	
>40	10	5.5	38	17.4	0.0002
Median, IQR	22	18-27	25	19-34	0.001
ALT, IU/dL, n, %					
≤40	158	86.3	144	66.1	
>40	25	13.7	74	33.9	<0.0001
Median, IQR	25	18-32	31	22-50	<0.0001

Table S7. Change from baseline to T1 (mean, 95% confidence interval) comparison between DOR-based and RPV-based regimens (men, n=401).

T1-T0	Doravirine N=183	Rilpivirine N=218	P-value*
BMI, Kg/m^2	-0.0 (-0.2, 0.2)	**0.1 (0.0, 0.2)**	0.65
Weight, Kg	0.2 (-0.3, 0.6)	**0.4 (0.0, 0.7)**	0.78
Total cholesterol, mg/dL	**-20 (-25, -14)**	**-16 (-20, -11)**	0.80
HDL-cholesterol, mg/dL	-1 (-2, 0)	**-2 (-3, -1)**	0.20
Total cholesterol /HDL-cholesterol	**-0.35 (-0.39, -0.20)**	-0.08 (-0.30, 0.13)	0.87
LDL-cholesterol, mg/dL	**-15 (-20, -10)**	**-8 (-12, -5)**	0.44
Triglycerides, mg/dL	**-24 (-40, -8)**	**-26 (-39, -12)**	0.12
**Not on lipid-lowering drugs**			
Total cholesterol, mg/dL	**-18 (-24, -13)**	**-17 (-21, -13)**	0.26
HDL-cholesterol, mg/dL	-1 (-2, 1)	**-3 (-4, -1)**	0.008
Total cholesterol /HDL-cholesterol	**-0.33 (-0.45, -0.22)**	-0.05 (-0.22, 0.13)	0.48
LDL-cholesterol, mg/dL	**-14 (-20, -9)**	**-9 (-13, -6)**	0.71
Triglycerides, mg/dL	**-24 (-44, -5)**	**-24 (-38, -10)**	0.19
**Liver enzymes**			**P****
AST ≤40 UI/L at T0	**2 (1, 4)**	**3 (1, 5)**	0.15
AST >40 UI/L at T0	-4 (-23, 15)	-3 (-14, 7)	0.83
ALT ≤40 UI/L at T0	**3 (2, 5)**	**10 (7, 13)**	0.001
ALT >40 UI/L at T0	-10 (-20, 1)	-4 (-14, 7)	0.72

ALT: alanine aminotransferase; AST: aspartate aminotransferase; DOR1: lamivudine/tenofovir disoproxil fumarate; DOR2: other DOR-based regimens; HDL: high density lipoprotein; LDL: low density lipoprotein

Bold: change from baseline p<0.05 (paired t-test).

*multivariate analysis of variance, including baseline age, weight, previous use of PI and INI, previous and current use of TAF and TDF, plus: -lipid lowering drugs use in the overall sample.

** multivariate analysis of variance, including baseline age, weight, previous and current use of TDF, hepatic virus coinfections.

Table S8. Change from baseline to T1 (mean, 95% confidence interval), comparison between men on 3TC/TDF/DOR (DOR1) or other DOR-based regimens (DOR2) (n=183).

T1-T0	DOR1 N=106	DOR2 N=77	P-value*
BMI, Kg/m^2	-0.1 (-0.2, 0.3)	-0.1 (-0.3, 0.2)	0.69
Weight, Kg	0.3 (-0.4, 0.9)	0.0 (-0.6, 0.7)	0.65
Total cholesterol, mg/dL	**-26 (-33, -18)**	**-11 (-19, -3)**	0.89
HDL-cholesterol, mg/dL	-1 (-3, 0)	-0 (-2, 2)	0.04
Total cholesterol /HDL-cholesterol	**-0.41 (-0.60, -0.22)**	**-0.26 (-0.48, -0.04)**	0.36
LDL-cholesterol, mg/dL	**-20 (-26, -14)**	-7 (-16, 1)	0.75
Triglycerides, mg/dL	**-19 (-36, -2)**	-30 (-62, 1)	0.94
**Not on lipid-lowering drugs**			
Total cholesterol, mg/dL	**-24 (-31, -17)**	**-8 (-17, -0)**	0.82
HDL-cholesterol, mg/dL	-1 (-3, 1)	-0 (-2, 3)	0.03
Total cholesterol /HDL-cholesterol	**-0.39 (-0.58, -0.21)**	-0.22 (-0.47, 0.03)	0.24
LDL-cholesterol, mg/dL	**-19 (-25, -13)**	-5 (-15, 4)	0.72
Triglycerides, mg/dL	**-18 (-35, -1)**	-36 (-80, 9)	0.92
**Liver enzymes**			**P-value****
AST ≤40 UI/L at T0	**2 (1, 4)**	**3 (1, 4)**	0.85
AST >40 UI/L at T0	-6 (-70, 59)	-3 (-21, 15)	0.74
ALT ≤40 UI/L at T0	**4 (2, 6)**	2 (-0, 4)	0.02
ALT >40 UI/L at T0	**-16 (-28, -4)**	-2 (-21, 16)	0.13

ALT: alanine aminotransferase; AST: aspartate aminotransferase; DOR1: lamivudine/tenofovir disoproxil fumarate; DOR2: other DOR-based regimens; HDL: high density lipoprotein; LDL: low density lipoprotein

Bold: change from baseline p<0.05 (paired t-test).

*multivariate analysis of variance, including baseline age, weight, previous use of PI and INI, previous use of TAF and TDF, plus: -lipid lowering drugs use in the overall sample.

** multivariate analysis of variance, including baseline age, weight, previous use of TAF and TDF, hepatic virus coinfections.
